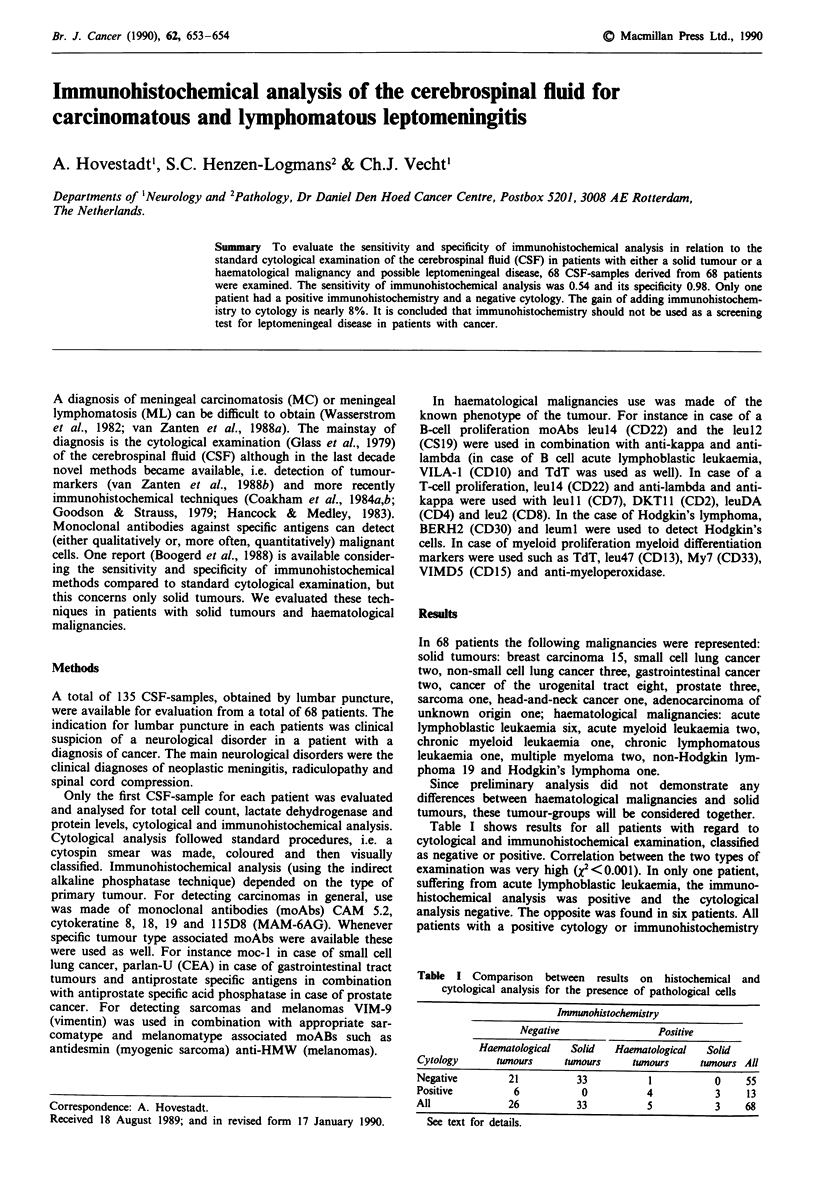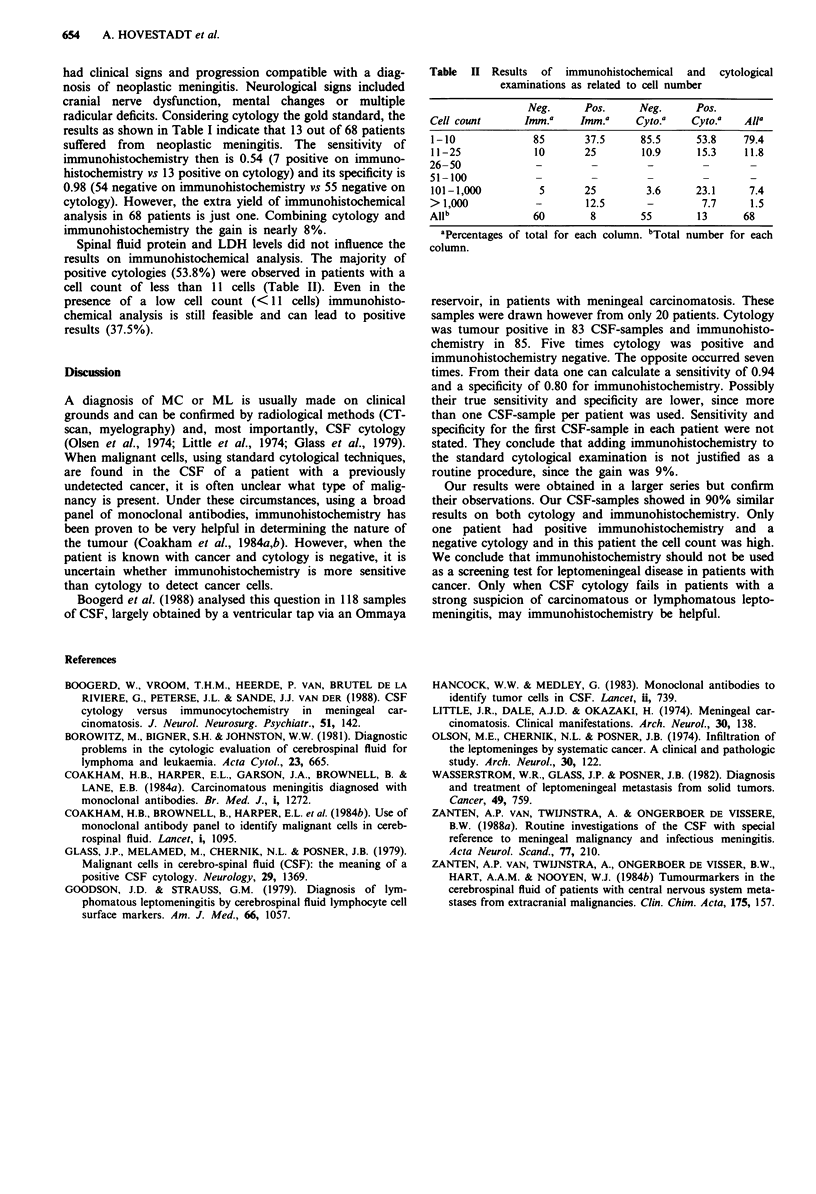# Immunohistochemical analysis of the cerebrospinal fluid for carcinomatous and lymphomatous leptomeningitis.

**DOI:** 10.1038/bjc.1990.349

**Published:** 1990-10

**Authors:** A. Hovestadt, S. C. Henzen-Logmans, C. J. Vecht

**Affiliations:** Department of Neurology, Dr. Daniel Den Hoed Cancer Centre, Rotterdam, The Netherlands.

## Abstract

To evaluate the sensitivity and specificity of immunohistochemical analysis in relation to the standard cytological examination of the cerebrospinal fluid (CSF) in patients with either a solid tumour or a haematological malignancy and possible leptomeningeal disease, 68 CSF-samples derived from 68 patients were examined. The sensitivity of immunohistochemical analysis was 0.54 and its specificity 0.98. Only one patient had a positive immunohistochemistry and a negative cytology. The gain of adding immunohistochemistry to cytology is nearly 8%. It is concluded that immunohistochemistry should not be used as a screening test for leptomeningeal disease in patients with cancer.


Br. J. Cancer (1990), 62, 653–654 © Macmillan Press Ltd., 1990

### Immunohistochemical analysis of the cerebrospinal fluid for carcinomatous and lymphomatous leptomeningitis

A. Hovestadt[1], S.C. Henzen-Logmans[2] & Ch.J. Vecht[1]

*Departments of [1]Neurology and [2]Pathology, Dr Daniel Den Hoed Cancer Centre, Postbox 5201, 3008 AE Rotterdam, The Netherlands.*

**Summary** To evaluate the sensitivity and specificity of immunohistochemical analysis in relation to the standard cytological examination of the cerebrospinal fluid (CSF) in patients with either a solid tumour or a haematological malignancy and possible leptomeningeal disease, 68 CSF-samples derived from 68 patients were examined. The sensitivity of immunohistochemical analysis was 0.54 and its specificity 0.98. Only one patient had a positive immunohistochemistry and a negative cytology. The gain of adding immunohistochemistry to cytology is nearly 8%. It is concluded that immunohistochemistry should not be used as a screening test for leptomeningeal disease in patients with cancer.

A diagnosis of meningeal carcinomatosis (MC) or meningeal lymphomatosis (ML) can be difficult to obtain (Wasserstrom *et al.*, 1982; van Zanten *et al.*, 1988*a*). The mainstay of diagnosis is the cytological examination (Glass *et al.*, 1979) of the cerebrospinal fluid (CSF) although in the last decade novel methods became available, i.e. detection of tumour-markers (van Zanten *et al.*, 1988*b*) and more recently immunohistochemical techniques (Coakham *et al.*, 1984*a*,*b*; Goodson & Strauss, 1979; Hancock & Medley, 1983). Monoclonal antibodies against specific antigens can detect (either qualitatively or, more often, quantitatively) malignant cells. One report (Boogerd *et al.*, 1988) is available considering the sensitivity and specificity of immunohistochemical methods compared to standard cytological examination, but this concerns only solid tumours. We evaluated these techniques in patients with solid tumours and haematological malignancies.

### Methods

A total of 135 CSF-samples, obtained by lumbar puncture, were available for evaluation from a total of 68 patients. The indication for lumbar puncture in each patients was clinical suspicion of a neurological disorder in a patient with a diagnosis of cancer. The main neurological disorders were the clinical diagnoses of neoplastic meningitis, radiculopathy and spinal cord compression.

Only the first CSF-sample for each patient was evaluated and analysed for total cell count, lactate dehydrogenase and protein levels, cytological and immunohistochemical analysis. Cytological analysis followed standard procedures, i.e. a cytospin smear was made, coloured and then visually classified. Immunohistochemical analysis (using the indirect alkaline phosphatase technique) depended on the type of primary tumour. For detecting carcinomas in general, use was made of monoclonal antibodies (moAbs) CAM 5.2, cytokeratine 8, 18, 19 and 115D8 (MAM-6AG). Whenever specific tumour type associated moAbs were available these were used as well. For instance moc-1 in case of small cell lung cancer, parlan-U (CEA) in case of gastrointestinal tract tumours and antiprostate specific antigens in combination with antiprostate specific acid phosphatase in case of prostate cancer. For detecting sarcomas and melanomas VIM-9 (vimentin) was used in combination with appropriate sarcomatype and melanomatype associated moABs such as antidesmin (myogenic sarcoma) anti-HMW (melanomas).

In haematological malignancies use was made of the known phenotype of the tumour. For instance in case of a B-cell proliferation moAbs leu14 (CD22) and the leu12 (CS19) were used in combination with anti-kappa and anti-lambda (in case of B cell acute lymphoblastic leukaemia, VILA-1 (CD10) and TdT was used as well). In case of a T-cell proliferation, leu14 (CD22) and anti-lambda and anti-kappa were used with leu11 (CD7), DKT11 (CD2), leuDA (CD4) and leu2 (CD8). In the case of Hodgkin's lymphoma, BERH2 (CD30) and leum1 were used to detect Hodgkin's cells. In case of myeloid proliferation myeloid differentiation markers were used such as TdT, leu47 (CD13), My7 (CD33), VIMD5 (CD15) and anti-myeloperoxidase.

### Results

In 68 patients the following malignancies were represented: solid tumours: breast carcinoma 15, small cell lung cancer two, non-small cell lung cancer three, gastrointestinal cancer two, cancer of the urogenital tract eight, prostate three, sarcoma one, head-and-neck cancer one, adenocarcinoma of unknown origin one; haematological malignancies: acute lymphoblastic leukaemia six, acute myeloid leukaemia two, chronic myeloid leukaemia one, chronic lymphomatous leukaemia one, multiple myeloma two, non-Hodgkin lymphoma 19 and Hodgkin's lymphoma one.

Since preliminary analysis did not demonstrate any differences between haematological malignancies and solid tumours, these tumour-groups will be considered together.

Table I shows results for all patients with regard to cytological and immunohistochemical examination, classified as negative or positive. Correlation between the two types of examination was very high ($\chi^2 < 0.001$). In only one patient, suffering from acute lymphoblastic leukaemia, the immunohistochemical analysis was positive and the cytological analysis negative. The opposite was found in six patients. All patients with a positive cytology or immunohistochemistry

**Table I** Comparison between results on histochemical and cytological analysis for the presence of pathological cells

| | *Immunohistochemistry* | | | | |
|---|---|---|---|---|---|
| | *Negative* | | *Positive* | | |
| *Cytology* | *Haematological tumours* | *Solid tumours* | *Haematological tumours* | *Solid tumours* | *All* |
| Negative | 21 | 33 | 1 | 0 | 55 |
| Positive | 6 | 0 | 4 | 3 | 13 |
| All | 26 | 33 | 5 | 3 | 68 |

See text for details.

Correspondence: A. Hovestadt.
Received 18 August 1989; and in revised form 17 January 1990.

had clinical signs and progression compatible with a diagnosis of neoplastic meningitis. Neurological signs included cranial nerve dysfunction, mental changes or multiple radicular deficits. Considering cytology the gold standard, the results as shown in Table I indicate that 13 out of 68 patients suffered from neoplastic meningitis. The sensitivity of immunohistochemistry then is 0.54 (7 positive on immunohistochemistry *vs* 13 positive on cytology) and its specificity is 0.98 (54 negative on immunohistochemistry *vs* 55 negative on cytology). However, the extra yield of immunohistochemical analysis in 68 patients is just one. Combining cytology and immunohistochemistry the gain is nearly 8%.

Spinal fluid protein and LDH levels did not influence the results on immunohistochemical analysis. The majority of positive cytologies (53.8%) were observed in patients with a cell count of less than 11 cells (Table II). Even in the presence of a low cell count (<11 cells) immunohistochemical analysis is still feasible and can lead to positive results (37.5%).

**Table II** Results of immunohistochemical and cytological examinations as related to cell number

| Cell count | Neg. Imm.[a] | Pos. Imm.[a] | Neg. Cyto.[a] | Pos. Cyto.[a] | All[a] |
|---|---|---|---|---|---|
| 1–10 | 85 | 37.5 | 85.5 | 53.8 | 79.4 |
| 11–25 | 10 | 25 | 10.9 | 15.3 | 11.8 |
| 26–50 | – | – | – | – | – |
| 51–100 | – | – | – | – | – |
| 101–1,000 | 5 | 25 | 3.6 | 23.1 | 7.4 |
| >1,000 | – | 12.5 | – | 7.7 | 1.5 |
| All[b] | 60 | 8 | 55 | 13 | 68 |

[a]Percentages of total for each column. [b]Total number for each column.

### Discussion

A diagnosis of MC or ML is usually made on clinical grounds and can be confirmed by radiological methods (CT-scan, myelography) and, most importantly, CSF cytology (Olsen *et al.*, 1974; Little *et al.*, 1974; Glass *et al.*, 1979). When malignant cells, using standard cytological techniques, are found in the CSF of a patient with a previously undetected cancer, it is often unclear what type of malignancy is present. Under these circumstances, using a broad panel of monoclonal antibodies, immunohistochemistry has been proven to be very helpful in determining the nature of the tumour (Coakham *et al.*, 1984*a*,*b*). However, when the patient is known with cancer and cytology is negative, it is uncertain whether immunohistochemistry is more sensitive than cytology to detect cancer cells.

Boogerd *et al.* (1988) analysed this question in 118 samples of CSF, largely obtained by a ventricular tap via an Ommaya reservoir, in patients with meningeal carcinomatosis. These samples were drawn however from only 20 patients. Cytology was tumour positive in 83 CSF-samples and immunohistochemistry in 85. Five times cytology was positive and immunohistochemistry negative. The opposite occurred seven times. From their data one can calculate a sensitivity of 0.94 and a specificity of 0.80 for immunohistochemistry. Possibly their true sensitivity and specificity are lower, since more than one CSF-sample per patient was used. Sensitivity and specificity for the first CSF-sample in each patient were not stated. They conclude that adding immunohistochemistry to the standard cytological examination is not justified as a routine procedure, since the gain was 9%.

Our results were obtained in a larger series but confirm their observations. Our CSF-samples showed in 90% similar results on both cytology and immunohistochemistry. Only one patient had positive immunohistochemistry and a negative cytology and in this patient the cell count was high. We conclude that immunohistochemistry should not be used as a screening test for leptomeningeal disease in patients with cancer. Only when CSF cytology fails in patients with a strong suspicion of carcinomatous or lymphomatous leptomeningitis, may immunohistochemistry be helpful.

### References

BOOGERD, W., VROOM, T.H.M., HEERDE, P. VAN, BRUTEL DE LA RIVIERE, G., PETERSE, J.L. & SANDE, J.J. VAN DER (1988). CSF cytology versus immunocytochemistry in meningeal carcinomatosis. *J. Neurol. Neurosurg. Psychiatr.*, **51**, 142.

BOROWITZ, M., BIGNER, S.H. & JOHNSTON, W.W. (1981). Diagnostic problems in the cytologic evaluation of cerebrospinal fluid for lymphoma and leukaemia. *Acta Cytol.*, **23**, 665.

COAKHAM, H.B., HARPER, E.L., GARSON, J.A., BROWNELL, B. & LANE, E.B. (1984*a*). Carcinomatous meningitis diagnosed with monoclonal antibodies. *Br. Med. J.*, **i**, 1272.

COAKHAM, H.B., BROWNELL, B., HARPER, E.L. *et al.* (1984*b*). Use of monoclonal antibody panel to identify malignant cells in cerebrospinal fluid. *Lancet*, **i**, 1095.

GLASS, J.P., MELAMED, M., CHERNIK, N.L. & POSNER, J.B. (1979). Malignant cells in cerebro-spinal fluid (CSF): the meaning of a positive CSF cytology. *Neurology*, **29**, 1369.

GOODSON, J.D. & STRAUSS, G.M. (1979). Diagnosis of lymphomatous leptomeningitis by cerebrospinal fluid lymphocyte cell surface markers. *Am. J. Med.*, **66**, 1057.

HANCOCK, W.W. & MEDLEY, G. (1983). Monoclonal antibodies to identify tumor cells in CSF. *Lancet*, **ii**, 739.

LITTLE, J.R., DALE, A.J.D. & OKAZAKI, H. (1974). Meningeal carcinomatosis. Clinical manifestations. *Arch. Neurol.*, **30**, 138.

OLSON, M.E., CHERNIK, N.L. & POSNER, J.B. (1974). Infiltration of the leptomeninges by systematic cancer. A clinical and pathologic study. *Arch. Neurol.*, **30**, 122.

WASSERSTROM, W.R., GLASS, J.P. & POSNER, J.B. (1982). Diagnosis and treatment of leptomeningeal metastasis from solid tumors. *Cancer*, **49**, 759.

ZANTEN, A.P. VAN, TWIJNSTRA, A. & ONGERBOER DE VISSERE, B.W. (1988*a*). Routine investigations of the CSF with special reference to meningeal malignancy and infectious meningitis. *Acta Neurol. Scand.*, **77**, 210.

ZANTEN, A.P. VAN, TWIJNSTRA, A., ONGERBOER DE VISSER, B.W., HART, A.A.M. & NOOYEN, W.J. (1984*b*) Tumourmarkers in the cerebrospinal fluid of patients with central nervous system metastases from extracranial malignancies. *Clin. Chim. Acta*, **175**, 157.